# Representation Learning for Fine-Grained Change Detection

**DOI:** 10.3390/s21134486

**Published:** 2021-06-30

**Authors:** Niall O’ Mahony, Sean Campbell, Lenka Krpalkova, Anderson Carvalho, Joseph Walsh, Daniel Riordan

**Affiliations:** 1 Lero—The Irish Software Research Centre, V92 CX88 Tralee, Ireland; sean.campbell@research.ittralee.ie (S.C.); lenka.krpalkova@staff.ittralee.ie (L.K.); anderson.carvalho@research.ittralee.ie (A.C.); Joseph.Walsh@staff.ittralee.ie (J.W.); Daniel.Riordan@staff.ittralee.ie (D.R.); 2 Department of Agricultural and Manufacturing Engineering, School of Science Technology Engineering and Maths (STEM), Kerry Campus, Munster Technological University, V92 CX88 Tralee, Ireland; 3 IMaR Research Centre, Kerry Campus, Munster Technological University, V92 CX88 Tralee, Ireland

**Keywords:** change detection, representation learning, latent space visualisation

## Abstract

Fine-grained change detection in sensor data is very challenging for artificial intelligence though it is critically important in practice. It is the process of identifying differences in the state of an object or phenomenon where the differences are class-specific and are difficult to generalise. As a result, many recent technologies that leverage big data and deep learning struggle with this task. This review focuses on the state-of-the-art methods, applications, and challenges of representation learning for fine-grained change detection. Our research focuses on methods of harnessing the latent metric space of representation learning techniques as an interim output for hybrid human-machine intelligence. We review methods for transforming and projecting embedding space such that significant changes can be communicated more effectively and a more comprehensive interpretation of underlying relationships in sensor data is facilitated. We conduct this research in our work towards developing a method for aligning the axes of latent embedding space with meaningful real-world metrics so that the reasoning behind the detection of change in relation to past observations may be revealed and adjusted. This is an important topic in many fields concerned with producing more meaningful and explainable outputs from deep learning and also for providing means for knowledge injection and model calibration in order to maintain user confidence.

## 1. Introduction

Change detection (CD), the process of identifying differences in object/phenomena over time/space, is often considered a fundamental low-level preprocessing step in many data analysis problems, such as in sensor data analytics, computer vision and process trend analysis. However, it can also be considered the primary task in many real-world applications such as remote sensing, surveillance, security and healthcare. The major challenge of CD is to separate real changes from false changes caused by different sensing conditions, e.g., sensor noise, suddenly varied lightings and camera movements in computer vision and unexpected changes in data distributions.

Most state-of-the-art CD methods assume real changes occur on a relatively large amount of data and are salient enough to transcend detailed changes caused by these factors. However, there are many applications where it is not feasible to collect data of sufficient breadth or depth for this method to be reliable, i.e., interactions between different combinations of conditions that were not accounted for at the design stage can induce variability that clouds and alters the characteristic features of significant changes, especially to each scenario. Clearly, for such scenarios, it is difficult for even the most modern deep learning techniques to generalise the features of changes of interest. This article will review the current state-of-the-art methods and some of the challenges to reliable detection of fine-grained change. In particular, we focus on techniques that can be applied to the representations learned by artificial intelligence in multi-task, multi-modal, open-set and online learning settings with little data to aid in navigating variability and uncertainty so that significant changes become apparent.

Representation Learning (RL) refers to the methodology of learning to represent data in the most simple form possible that preserves the details relevant to the task(s) at hand. RL is an integral part of many machine learning algorithms and comes in different guises but all essentially have the common goal of defining a feature space in which we can make observations on the relation between entities. This objective is an important one with many practical applications, which we survey in Section 2. In Section 3, we give some context of how RL has come to be at the forefront of the state-of-the-art in change detection with some historical background from its statistical origins to the advent of deep learning. We then examine the different ways in which change can be presented in RL frameworks, followed by a comparison of the different types of architecture, including metric learning, generative models and graph neural networks and a breakdown of the common techniques for manipulating their latent feature space to produce change representations that offer better interpretability and discriminatory capability, all in Section 4. Lastly, in Section 5, we review some gaps in the research towards extending RL to change detection use cases, including online learning, handling heterogeneous data and explaining the reasoning of a model.

## 2. Applications of Change Detection

Change detection is quite a broad term that encapsulates anything from low-level processes in algorithms such as edge detection to high-level tasks that must employ contextual understanding to determine significant change. This section will review applications of the latter, which include methods for detecting differences on a spatial scale, on a time scale, on triggered objects or on some hybrid of these types.

In many of these applications, it is sometimes desirable to distinguish instances of change by capturing slight and subtle differences. For instance, it may be desirable to track the trend of continuous change in the recent past (e.g., to track the progression of a disease [1]) for each instance. It is also often necessary to accommodate intra-class variation for a CD system to be effective in its intended application, i.e., in applications such as biomedical diagnosis and all-important buildings (e.g., dam) monitoring, it is critical to guarantee detection sensitivity and accuracy of minute changes in each observation by taking measures to maximise the signal-to-noise ratio by adapting our reasoning specific to the class of object we are looking at.

This practice is known as fine-grained (FG) data analysis, which targets the study of objects/phenomena from subordinate categories, e.g., if the base task is to detect changes in human health, the FG task may be to detect changes specific to a specific person. FG analysis is a long-standing and fundamental problem because small inter-class variations in the phenomenon of interest can often be masked by large intra-class variations de to ancillary data [2]. However, it is an important problem and has become ubiquitous in diverse CD applications such as automatic biodiversity monitoring [3], climate change evaluation [4], intelligent retail [5], intelligent transportation [6], and many more.

### 2.1. Remote Sensing

Remote sensing (RS) is the collection of images of an object/area from afar, typically from a satellite or aircraft and usually of the Earth’s surface. CD is an important aspect of RS as a tool to reliably quantify spectral differences in the radiation received from features of interest, whether it be for the study of spatial differences in surveying applications such as land use and land cover classification [7], agricultural analyses [8], environmental monitoring [4], disaster assessment [9] and map revision [10].

Handling uncertainty is one of the main concerns in these applications as many external factors, such as sensor gain (random error due to imperfect calibrated camera sensor arrays), image noise and atmospheric conditions [11] influence the absolute sensor readings, which means that corresponding subtle differences between images, even in the same location, in the large datasets, which are typically accrued, is not so straightforward. Specialised CD techniques for addressing this concern include fuzzy logic, Monte Carlo analysis and geostatistical analysis [12].

These methods employ different forms of reasoning in order to take into account uncertainty present even in “ground truth” data. Fuzzy logic employs membership functions to express the vagueness of labels (e.g., land cover may vary continuously in transition zones), thus fuzzy classes are assigned in proportion for each entity and some ambiguity is mitigated. Uncertainty due to human error during the manual labelling has also been taken into account by explicitly incorporating label jitter (inconsistencies in labelling near class boundaries arising from human error in the annotation process) into the model training process in the form of an activity boundary smoothing method that explicitly allows overlapping activity labels [11]. The Monte Carlo method is a paradigm that has to do with randomness—a random sample, drawn from the error probability distribution of each measurement, is added to that measurement, and the net effect on the overall picture is stored. This procedure is repeated several hundred times and the resulting collection of maps is analysed to see how measurement uncertainty has propagated to the outcome. If many of the maps show a large variation in a measurement at a particular location, then we know there is a lot of uncertainty. Lastly, geostatistics can also be useful in improving measurements in remote sensing through the use of statistical understandings of spatially varying properties. For example, the expected range of the difference in measurements over a region (described in what is called a variogram) is directly related to texture and/or object size. Bayesian inference is also often applied in geostatistics to interpolate the value of a random field (e.g., the elevation, z, of the landscape as a function of the geographic location) at an unobserved location from observations of its value at nearby locations.

Terrestrial based mapping applications also apply such CD techniques to overcome uncertainty arising from large sudden changes in camera pose, dynamic objects (i.e., objects that can be removed from a scene and thereby affect its appearance) and limited field of view. Three-dimensional sensing has become very popular for aiding in overcoming some of these challenges as recently, sensors have become available that can provide reliable depth information for each pixel. These sensors allow the physical geometry of objects to be measured with relative immunity to illumination variations and perspective distortions, which enables simple geometric comparisons of extracted 3D shapes with simulated reference shapes to be effective for change detection [13]. Challenges in this area include misalignment in point cloud registration and designing algorithms efficient enough to compensate for the increasing data volume.

### 2.2. Video Surveillance

In simple computer vision applications, where the sources of uncertainty can be constrained (e.g., in industrial manufacturing lines where lighting and environmental conditions are well controlled), CD techniques such as edge detection in images are a powerful tool. For example, high precision industrial vision/sensing systems for the inspection and categorisation of objects can achieve accuracies well within the allowable tolerance of standard measurement instruments automatically, non-invasively and without requiring precise fixturing with the aid of high-resolution cameras, a lot of specialised knowledge in machine vision and edge detection [14] and sub-pixel detection techniques [15].

The most common use cases of more complex applications of CD in video surveillance to date entail abnormal changes of foreground human behaviours/activities that could pose damage or danger to human properties and lives, e.g., fall detection [16], aggressive/violent behaviour detection [17] and pedestrian intention estimation for advanced driver-assistance systems (ADAS) [18]. These applications require change-detection to happen in real-time and in unregulated environments (environments where variables such as lighting conditions, camera pose, object pose and object characteristics are relatively ill-constrained compared to industrial/laboratory conditions). The challenges associated with these requirements are discussed further in Section 5.1.

### 2.3. Healthcare

CD is an extremely common task in the healthcare sector since medical diagnoses are essentially based on the difference between a patient’s state and known “healthy” conditions or their previous state. Scientists are now trying to automate some of these processes to relieve some of the burden on the medical sector arising from the demographically older population and enable more ubiquitous and personalised remote healthcare solutions. Some of this research investigates the use of wireless sensors for monitoring the physiological profile of the wearer in a continuous, real-time, and non-intrusive manner for the early detection of illness/incident [19,20]. Continuous monitoring involves the recognition of complex patterns across a wide variety of scenarios, e.g., as patients make lifestyle changes during recovery, and fine-grained analysis as each patient will behave differently [21]. It is also desirable to perform CD on the edge (i.e., for the algorithms to be processed on or close to the sensor in an Internet of Things network) to mitigate the need for raw data to be transmitted and save bandwidth but more importantly where real-time data processing and decision making are important for closed-loop systems that must maintain critical physiological parameters [22]. The reduced processing and memory capability of hardware on the edge necessitates for algorithms to be lightweight and efficient. Maintaining CD performance in the face of problems deriving from changes in data distribution over time is also a challenge for which distributed learning systems are a promising proposition. This is where each edge node implements part of a common neural network and exchanges weights with other peer nodes, and this framework can efficiently deal with covariate shift as only the device containing the first layers of the network has to be modified.

CD algorithms also play an important role in diagnostic fields involving signal analysis such as cardiology [23] and the analysis of medical images, e.g., in retinopathy and radiography [1,24]. CD also has applications in sensor-assisted/robot-assisted surgery in the analysis of data from sensors for detecting changes in tissue characteristics [25].

### 2.4. Monitoring Man-Made Systems

Complex computer-based systems aimed to assist/automate tasks that consist of multiple interconnected components take considerable effort to maintain. The monitoring and alerting of changes to the procedures within these systems is of great importance to ensure no alterations made during system maintenance interfere with critical functions. Examples where CD has been implemented include clinical decision support systems [26], web ontologies [24] and safety-critical software [27].

The modelling of dynamic systems can also be considered an application of CD principles, e.g., in the detection of sensor and actuator failures [28] and the tracking of manoeuvring vehicles/robots [29]. System dynamics endeavours to derive a mathematical model of the non-linear behaviour of complex systems in order to understand and track them effectively. In practice, these models not only have to reflect the behaviour of the system but must also accommodate deficiencies in the sensing hardware used to monitor it. For example, some models account for measurement drift by appending a second-order term that describes the characteristic behaviour of the sensor between calibrations [30] while others learn the interaction between the system and sensor(s) as a whole with a neural network [28]. In addition, abrupt sensor faults can be addressed by sampling over a longer time window when training such a neural network [28].

## 3. History of Change Detection

In this section, we will give a brief overview of the evolution of the tools available in the field of CD. As these tools progressed, the size, dimensionality and complexity of the data the algorithms were capable of processing also progressed. Methods initially focused on univariate time series data that followed parametric assumptions and then began learning non-linear relationships in non-parametric sequential data with machine learning, eventually being able to model multivariate, non-stationary data and finally were able to process high-dimensional computer vision data with deep learning.

### 3.1. Statistical Methods

Early research in CD was concerned with change point detection in sequential data. The main application area for this research was industrial statistical process control (SPC), where the approach is to detect the changes in the mean of the time series, assuming the baseline process to be stationary and the shift pattern to be a step function that is sustained after the shift. The theory behind change point detection is known as sequential analysis. Some notable methods include Seasonal Trend Decomposition using LOESS (Locally Estimated Scatterplot Smoothing) [31] and PELT (Pruned Exact Linear Time) algorithm [32]. STL decomposes the time series into three components: trend, season and residual where the rate of change and smoothness of the season and trend, respectively, can be tuned to the periodicity of the input data. PELT, a more recent algorithm, uses a cost function to minimise the computational cost of detecting the optimal number of change points. These algorithms have been integrated into many programming frameworks [33] and are efficient and non-parametric but require fine-tuning to be effective, e.g., PELT requires the penalty to be adjusted to prevent over/underfitting.

Slightly more powerful statistical CD schemes for non-parametric problems are based on generalised likelihood ratio statistics [34], which assume that signal patterns follow a known distribution during “normal” conditions and deviation from this distribution is distinguishable and is an indicator that a change has occurred. These methods are far more “automatic” in that they do not require manual oversight or tuning. A classic example is the Conventional Cumulative Sum (CUSUM) algorithm, which monitors the correlation of signal patterns with, for example, a Gaussian distribution with mean μ and known standard deviation σ, and accumulates deviations from these statistics until they reach a certain threshold. If the threshold is reached within a predefined time window then a change has been detected [35]. Some variants of CUSUM are also able to handle non-stationary sequences (where the “normal” distribution can shift) [36] and FG risk adjustment (by replacing static control limits with simulation-based dynamic probability control limits for each subject) [37].

In applications where data may be subject to a variety of sources of variation that influence the distribution of occurrence of particular phenomena (e.g., long-term periodic signal variation due to the day of the week/time of day, etc.), the source of deviations may be accounted for and recognised so as not to falsely trigger real anomalies. However, models become increasingly complex the more exclusions it has to accommodate and it is often not possible to identify all possible sources of noise during system design. Therefore, algorithms must be able to automatically learn to differentiate noise from natural signal variation in a wide variety of scenarios with limited information. This class of algorithm is known as machine learning, of which early methods used techniques such as Gaussian Mixture Models, which represent signal relations as probability distributions and compare them against each other [37], or kernel functions and later work, which took advantage of the acceleration of machine learning with parallel processing, which we will cover in the next section.

### 3.2. Deep Learning

Recently, there has been a big jump in our ability to recognise complex features thanks to a development called deep learning (DL), and more specifically, the neural network (NN) computing architecture, which emulates the theorised functioning of the human brain. The adjective “deep” is often assumed to mean that the architecture consists of many layers of computing cells, sometimes called “neurons”, that each perform a simple operation. The result of each computation being an activation signal that is passed through to the neurons in proceeding layers. Each neuron assigns a weight to each of its inputs and adds a bias value if necessary. By tuning these weights and biases, a model can be trained/learned to capture “deeper” local information and features through exploiting self-organisation and interaction between small units [38]. It is also for this reason that deep neural networks (DNNs) are often computed using GPUs, or similar hardware suited to matrix multiplication, and the availability of such computing resources is what has fuelled the recent activity and great strides in the predictive capability of artificial intelligence.

The power of DL comes at the cost of the need for large amounts of data to learn from. In terms of whether this data requires manual labels, most deep learning approaches can be grouped into supervised and unsupervised methods. Supervised methods can generalise better but only where large annotated datasets are available, which for less popular applications such as CD and FG recognition is not that common. However, there are many methods for training DL models in such circumstances, in both supervised and unsupervised settings [10], including one-shot learning, generative-adversarial learning and structure/theory-based methods, which will be expanded upon in this review.

## 4. Representation Learning for Fine-Grained Change Detection

The main function of RL is to encode higher-order statistics of convolutional activations/features learnt by a DNN to enhance the mid-level learning capability, i.e., the focus is on enhancing the intermediate feature descriptor learned by a DL model to output a “good” representation of the input data. This field has become an important research track in the area of machine learning, intending to provide more informative numerical representations of the observed data. Naturally, progress in this field is applicable in FG CD applications also, as good representations provide a means of discrimination based on intrinsic data properties while also determining the relation between entities. In this section, we will discuss training regimes, ways of representing change and ways of communicating/interacting with change representations concerning the applications discussed in Section 2.

### 4.1. Change Representation

The motivation for the task of learning to represent change lies in the fact that in some cases, humans can distinguish and approximate subtle differences from one instance to the next quite easily regardless of the domain with very little training. This begs the question, how does the brain detect change? Researchers found that the longer participants are exposed to the initial texture, the faster their reaction time and ability to identify the changes [39]. This implies that our ability to detect change relies on our becoming familiar with a baseline pattern and compartmentalising that familiarity, allowing the raw data pertaining to the normal state to be processed in the background, and more effort may be allocated to noticing deviations from normal conditions.

Most change representations (CR) contain two elements. The first element is some description of the discriminative visual features between normal and changed samples and the second is some means of classifying/quantifying the change between samples. For the first element, many vision-based applications, particularly in RS, just produce a visual output, called a change map, of which pixels have changed between the corresponding images of two datasets [10]. Additional information concerning the second element of CR can be encoded as binary, triple (i.e., positive, negative or no-change) or type indicators of change for each pixel in these change maps [13]. Most non-visual applications (and applications that do not just have visual information) work with a propositional feature vector representation of data. This means that each instance is represented as a vector and the components of the vector are either binary, nominal or numerical indicators of what features are present in the input data. Both first and second elements of CR are contained within this feature vector because (a) the distinguishing features are learnt using machine learning and (b) the vectors are structured such that some means of measuring the similarity between vectors may be used to classify the type and degree of change. This notion of similarity may be a distance measure, a graph encoding or the result of any vector or graph operator between feature vectors. We will examine the frameworks for encoding these relationships between representations in the subsequent subsections.

In a supervised setting, each CR requires labelling. The number of labelled training samples required to train a model with sufficient generalisability is dependent on the numerosity, complexity and consistency of the various interactions between the components of the projected feature vector. It is also necessary for this training dataset to be balanced, i.e., the dataset must contain IID (independent and identically distributed) samples of data and their associated labels so that probabilistic models do not favour classes with the largest proportion of observations. These dataset management requirements are especially difficult to manage in online learning, domain transfer and open-set classification problems where new objects must be recognised without impairing performance on previous old objects [40].

#### 4.1.1. One-Shot Learning

One way around the issues of dataset management and online model retraining is to be able to learn from fewer data so that when new objects/classes are observed, they may be recognised the next time they are observed. This is a challenging machine learning problem and is often referred to as zero/one/few-shot learning where objects must be distinguished as belonging to distinct classes based on zero/one/few previous observations, respectively. In this setting, learning is based on similarity rather than label assignment, and the training set X considers pairs/triplets of samples at a time, splitting them according to their similarity into the sets

$$\begin{array}{cc} S & =\{\left(x_{i},x_{j}\right)\in X\times X:x_{i}\;\mathrm{and}\;x_{j}\;\mathrm{are}\;\mathrm{similar}\},\\ D & =\{\left(x_{i},x_{j}\right)\in X\times X:x_{i}\;\mathrm{and}\;x_{j}\;\mathrm{are}\;\mathrm{dissimilar}\},\\ R & =\{\left(x_{i},x_{j},x_{l}\right)\in X\times X\times X:x_{i}\;\mathrm{is}\;\mathrm{more}\;\mathrm{similar}\;\mathrm{to}\;x_{j}\;\mathrm{than}\;\mathrm{to}\;x_{l}\}. \end{array}$$

The way in which the pairs/triplets (most methods sample triplets and therefore we will hereafter refer to the samples as triplets) are split is dependant on the end task, i.e., on whether we want to do classification, ranking or regression. In a classification setting, similar pairs in *S* belong to the same class and dissimilar pairs in *D* do not. In ordinal classification/ranking, the degree of similarity can be considered based on the ordering of the classes in *R*. In metric regression, similarity or dissimilarity (in the sets *S* and *D*) may be based on proximity to a target value within a certain margin.

This concept of having a set of datasets rather than there being one large dataset is central to the implementation of one-shot learning. Each set can contain relatively few annotated examples per class, which alleviates requirements for a large balanced dataset. Machine learning architectures that can learn from this data include meta-learning, manifold learning and metric learning. These architectures will be described further in Section 4.2, but in overview, these methods ensure more a reasonable speed of convergence compared to conventional deep learning by utilising some way of enforcing consistency between the outputs of training batches. This consistency may be gained by mapping the feature vector outputs to a latent space/manifold where the relative distance between feature vectors is regularised in metric/manifold learning or by using techniques such as episodic memory replay in meta-learning.

#### 4.1.2. Graph Embedding

Of course, it is not always possible for propositional features to encode all the knowledge available in the original data. However, knowledge graph embedding is an effective yet efficient way of converting feature vector representations into a low dimensional space in which the graph structural information and graph properties are maximally preserved. This can be hugely advantageous as embedding a graph $G=\left(V,\;E\right)$ with node data points $v_{a}\in V$ and edge similarities E∈V×V, that reflect auxiliary information and relationships between entities can reveal interesting properties that cannot be seen otherwise [41]. Graphs can be directed or undirected (i.e., edge relationships act in one direction only or both directions), sparse or dense (i.e., the number of edges is close to the minimal or the maximal number of edges) and connected or disconnected (i.e., there is a path from any point to any other point in the graph or there is not). Furthermore, relations can be represented as operations in the vector space, e.g., vectors, matrices, tensors, multivariate Gaussian distributions or even mixtures of Gaussians. The embedding process involves a scoring function to measure the plausibility of each relation on which optimisation is performed to maximise the total plausibility of the graph and mitigate data sparsity [42].

#### 4.1.3. Unsupervised Learning

In unsupervised representation learning, the gist is to learn an underlying low-dimensional subspace in which the geometric distances between the majority of the observed data are preserved.

Self-supervised learning is an unsupervised learning approach that involves changing or holding back certain aspects of the data in some way and training a model to predict or generate aspects of the missing information. A common workflow is to train a model on one or multiple pretext tasks (usually to reproduce an input image in a different context, the context being provided by a separate reference image) and then feed the mid-level latent representations of this model to fine-tune a simple model for the downstream task (e.g., change detection). For example, the authors of [43] have demonstrated this workflow in a CD application by enrolling a generative temporal prediction model to predict what a scene would look like at a given time-step and then compare the result with the actual image when that time comes about at runtime. Any deviation from the generated image is taken to be an indicator of a deviation from the natural expected sequence of events and a change detection is triggered. Most generative models have the goal of creating diverse and realistic images, but they can also be used as a specialisation of self-supervised representation learning were the goal is producing good features generally helpful for many tasks. Self-supervised learning offers an appealing alternative to supervised learning in that they are trained to model the full data distribution without requiring any modification of the original data. This field can be quite powerful in change detection without any prior knowledge and with few data samples because generative augmentation techniques can be used to learn representations that are invariant to augmentation signatures [44].

### 4.2. Types of Representation Learning Architectures

Here we discuss the base feature extractors that may be used to learn change representations. The choice of the format of change representation (discussed in Section 4.1) determines what architecture is most appropriate for learning to generate representations.

#### 4.2.1. Meta-Learning

Meta-Learning or “learning to learn” refers to the power to adapt previous learning experience to new, unseen, small data. Most of the techniques reviewed in this article could be classified as some form of meta-learning; however, the label is generally used for gradient-based approaches. The most representative of such techniques is MAML, where the focus is to meta-learn the best initialisation of parameters for a task learner. In this way, the perspective is switched from learning how to perform on data to learning to perform tasks. The learnt model assumes a task structure that incorporates exploitable meta-knowledge, i.e., a model that meta-learns would learn to bind data representations to their appropriate labels regardless of the actual content of the data representation or label, and would employ a general scheme to map these bound representations to appropriate classes or function values for prediction [45].

Some of these techniques can be prone to catastrophic forgetting, i.e., if we were to retrain the model again on the new data we want to accommodate in our task, we can indeed, for example, learn to recognise the features of the new object; however, the tuning done to the weights and biases has no regard for the other tasks we have trained the model to do already and so interferes with previously learned knowledge. This problem has been approached with many techniques, including regularisation (which imposes constraints on the update of the neural weights), dynamic architectures (e.g., collections of sub-networks and denoising autoencoders) and complementary learning systems and memory replay (which emulate the interplay of episodic memory and semantic memory in the human brain with networks that take both as input) [46].

#### 4.2.2. Metric Learning

**Figure 1:**
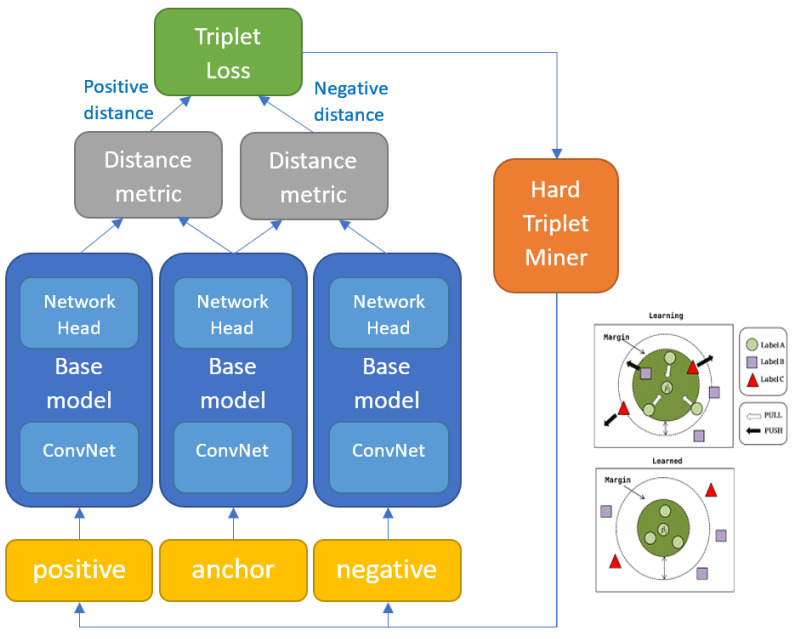
A triplet-based metric learning architecture. Each of the three samples is passed through the same embedding network, and the loss function determines how to space them apart in latent space.

As change detection is essentially the detection of differences observed in objects/phenomena, it is natural that distance/similarity-based machine learning solutions be suited to this task. Distance metric learning (DML) is a similarity-based machine learning method where data slices are passed pairwise through a siamese/triplet/quadruplet network, which is optimised to produce projections to a latent space with some notion of distance, such that similar samples are placed close together and dissimilar ones far apart. The notion of distance between the feature vectors projections (also referred to as embeddings) arises from the use of some distance metric (e.g., Euclidean distance) in the loss function implemented during training (as illustrated in Figure 1).

There are many types of loss function, the simplest being contrastive loss

$$\begin{array}{c} \ell^{\mathrm{contrast}}\left(i,\;j\right):=y_{ij}D_{ij}^{2}+\left(1-y_{ij}\right){\left[\alpha -D_{ij}\right]}_{+}^{2}. \end{array}$$

where $y_{ij}$ serves as a binary indicator of pair similarity, if a set of inputs belong to the set *S* (i.e., are positive pair), $y_{ij}=1$, and the loss function minimises the distance between their associated feature vectors ($D_{ij}$) and when input pairs belong to the set *D* (i.e., are dissimilar/negative), the loss function maximises $D_{ij}$ until they are at least a margin α apart. Iterations of loss functions to proceed contrastive loss (triplet loss, angular loss, margin loss, N-Pairs loss, also known as, InfoNCE, multi-similarity loss, tuplet margin loss and circle loss) introduce additional features such as placing fewer restrictions on the embedding space and allowing the model to account for variance in interclass dissimilarities [47]. For example, triplet loss:

$$\begin{array}{c} \ell^{\mathrm{triplet}}\left(a,\;p,\;n\right):={\left[D_{ap}^{2}-D_{an}^{2}+\alpha \right]}_{+}. \end{array}$$

merely tries to keep the distance to positives *p* smaller than the distance to negatives *n* for every anchor *a*, which means a constant margin α does not need to be selected (just a minimum one). It also has the consequence that the embedding space can be arbitrarily distorted, i.e., that visually diverse classes are embedded over a wider space than similar ones.

The advantages of metric learning algorithms include: (1) they are very simple and easy to implement; (2) they are usually efficient in space and time complexity; (3) they are often theoretically guaranteed [48]. The primary advantage comes from the high recognition capacity of the deep base model. DML is essentially a way of deploying DNN in an instance-based fashion, enabling remarkable FG recognition performance. However, the high dimensionality of the intermediate features can make it impractical for realistic applications, especially for the large-scale ones [2]. A critical element of metric learning is the selection of triplets during training (sometimes referred to as triplet mining, see Figure 1 for position in network). Triplets need to be selected such that consecutive batches vary in a gradual way so that the network can actually learn between batches but also such that all the important variations within the dataset get encountered with enough frequency during training so that the model can captures that variability. Different mechanisms for triplet mining exist. For example, hard triplet mining selects the most difficult triplets for each anchor. The difficulty of triplets is determined by running the most recent model at each training iteration to get the distance of all positive and negative embeddings for a set of anchors. Hard triplet mining selects the most distant embedding in *S* and the least distant embedding in *D* for each anchor. Since it is computationally infeasible to aggregate loss over all $O(n^{3})$ triplets and hard triplets can cause models to collapse, heuristics are used to speed up convergence. A well-known miner is semi-hard negative mining, which samples anchors and positives in batches from X and *S*, and finds the closest negatives within the batch further away than D(a, p) [49].

#### 4.2.3. Deep Generative Models

Deep generative models have a type of architecture where the output is some transformation of the input with the same dimensionality. Needing only a desired image output as a target to generate, this architecture can leverage smart training techniques to learn from a huge amount of data that are not extensively annotated. This is one of the main reasons why generative models have made large strides in our ability to successfully model complex, high-dimensional data in applications such as image generation [50], video generation [51] and point cloud completion [52] and why they have been implemented in many applications related to CD, including one-shot learning [53] and image interpolation [54].

The training technique may be direct (i.e., comparing the true and the generated probability distributions) or indirect (i.e., adversarial training where a discriminator network downstream from the generator network has the task of discriminating between ground truth and generated data and it is the generator’s job to fool the discriminator) [55].

**Figure 2:**
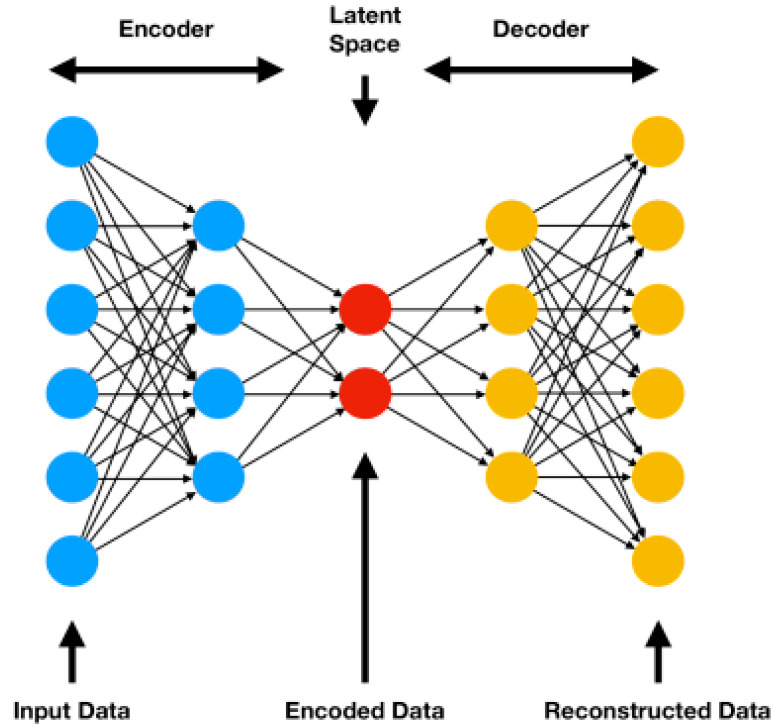
The autoencoder architecture can be considered a form of representation learning where the mid-level encoded data are interpreted as output. Reproduced with permission [60].

The most well-known of the former direct comparison techniques is the variational auto encoder (VAE), a technique that can model high-dimensional data flexibly to produce low-dimensional embeddings. The “variational” in VAE comes from a concept called variational inference, which refers to a technique for approximating probability densities through machine learning. “Auto” refers to the automatic regularisation of encoder embeddings during training and “encoder” refers to a type of neural network that produces a new feature representation from a set of input features. Similar to the generator-discriminator principle described in the last paragraph, the VAE adopts an encoder–decoder architecture (see Figure 2), where the encoder produces some distribution over the latent space and the decoder reverses the process on a sample of the encoding to produce something close to the input/target as closely as possible using an iterative optimisation process that can be trained by gradient descent to minimise the reconstruction/generation error. In applications where the encodings that reside in the mid-level latent space between encoder and decoder are taken as the output, VAEs can be considered to be a form of representation learning in that the same transformations and theory in geometric/information calculus may be applied to the latent space but gradient descent and deep learning is harnessed differently through the probabilistic nature of latent space distributions and the regularisation of these distributions using Kullback–Leibler divergence [56]. VAEs are considered to be more flexible than metric learning but less interpretable although there are techniques for interpreting representations coming to the fore [57]. For example, transformer networks utilise the mechanism of attention to indicate where in an input image salient activation occurs [58,59].

The latter form (indirect generative models) are known as generative adversarial networks and have many advantages, including being able to learn spatial relations and temporal correlations from target data and the ability to synthesise more training samples [61]. Generative techniques feature in many recent RL methods for this reason and can be very interesting in FG change detection applications where pattern discovery is essential to every new case. For instance, they have been integrated into the training procedure of a one-shot learning framework by [62] and have been used to generate augmentations for unsupervised anomaly detection [44].

As we have mentioned, generative models operate on the principle that distributions can be learned from data. However, it is often hard to understand and interpret the resulting embeddings and guide the representations with some downstream task in mind, i.e., to create data from noise. To this end, the recent state-of-the-art propose techniques such as bipartite attention between the transformer features and a selection of latent variables [58] and inconsistency loss for measuring the degree to which the model violates the assumptions on an adversarially-generated set of examples [63].

#### 4.2.4. Geometric Deep Learning

Graphs are used extensively in applied science as a way of organising data that prioritises certain patterns so that relationships between interacting features can be efficiently computed, stored and accessed. Recent years have seen a surge in approaches that automatically learn to encode graph structure into low-dimensional embeddings, using techniques based on deep learning and non-linear dimensionality reduction to leverage this information within graphs. This emerging domain is known as geometric deep learning (GDL) [64]. The authors of [65] provide a conceptual review of key advancements in the area of learning graph representations, which include matrix factorisation-based methods, random-walk based algorithms, and graph neural networks (GNNs). Of these techniques, GNNs are attracting the most interest as natural generalisations of convolutional networks to non-Euclidean graphs. i.e., graph convolutions are similar to the vanilla convolutions of CNNs except instead of operating on a grid, they operate on neighbouring nodes. This difference means that the numbers of nodes connections vary, and the nodes are unordered. There are two types of graph convolutions: spectral and spatial. Spectral convolutions consider graph representations as signals and operations such as the Fourier transform and other signal processing techniques to aggregate node information. Spatial methods represent graphs using pseudo-coordinates and use operations such as message passing to aggregate information between nodes. The former resembles vanilla convolutions mathematically and the latter conceptually. They both require some method of characterising the neighbourhood of each node with the use of eigendecomposition (the factorisation of a matrix into its eigenvalues and eigenvectors) or other related operations.

**Figure 3:**
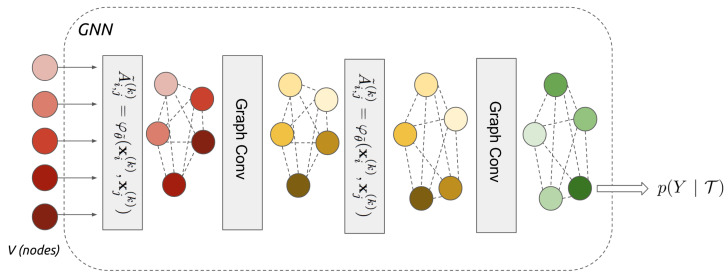
Graph neural network for representation learning. Note: dotted lines indicate learnt edge features and node colour changes indicate the aggregation of information by convolutional layers. Reproduced with permission [67].

The simplest of these operations is adjacency learning, which is useful in applications where the input set is believed to have some geometric structure, but the metric for measuring the geometry is not known a priori. For example, the GNN shown in Figure 3 generalises to learn edge features ${\tilde{A}}^{\left(k\right)}$ before every convolutional layer:

(1) $${\tilde{A}}_{i,j}^{\left(k\right)}=\varphi_{\tilde{\theta }}\left(x_{i}^{\left(k\right)},x_{j}^{\left(k\right)}\right)\;,$$

where φ is a symmetric function parametrised with, e.g., a neural network or decoder-encoder architecture, which learns a non-linear combination of the absolute difference between the individual features of each pair of nodes. GNNs contain relatively few layers (only two adjacency-convolutional layers are used in Figure 3) compared to CNN as the graph structure means fewer convolutions are required to share information between all nodes/regions of the input data.

Another important operator is the Laplacian operator Δ, which measures how a function changes “on average” as you move away from a given point. As will be discussed in later sections of this article, this divergence-based operator plays a key role in the analysis of manifolds and, in the context of GDL, Laplacian eigenfunctions generalise the classical Fourier bases, allowing spectral analysis to be performed on graphs [64]. For simple undirected graphs $G=\left(V,\;E\right)$, the graph Laplacian

(2) $$L_{G}=\sum_{(i,j)\in E}\left(e_{i}-e_{j}\right){\left(e_{i}-e_{j}\right)}^{⊤}$$

which can be denoted

(3) $$L_{G}=D-A$$

where *A* is the adjacency matrix and *D* is the degree matrix, a diagonal matrix that contains information about the degree of each vertex—that is, the number of edges attached to each vertex. For other types of graphs, there exist generalisations of $L_{G}$, such as the random-walk graph Laplacian for large graphs [65] and the Laplace Beltrami operator for manifolds [66]. Thus, it is commonly used in many fields where a link needs to be drawn between discrete representations, such as graphs, and continuous representations, such as vector spaces and manifolds.

An important property of graph operators is that they are symmetric, i.e., their output given in arguments is the same regardless of the order of the arguments, and positive semidefinite, i.e., that their eigenvalues are non-negative, which is important for facilitating efficient optimisation of complex higher rank matrices. Using local operators of graphs offers a powerful balance between expressivity and complexity of representations while also exploiting stationarity, connectivity and compositionality in the same way CNNs do [64].

GDL is being deployed in more and more applications by applying a graph structure to data, e.g., the state-of-the-art in change representation from electronic health records with missing values was achieved by creating nodes for medical concepts and implying connections among thousands of these concepts with a hybrid of VAE and GNN architectures that harnesses the qualities of graph representation and variational inference [68]. In the context of change detection, GNNs have also been used for change-point detection in multivariate time series with changeable correlation structure [69], in the unsupervised analysis satellite image time series [70], in contagion dynamics [71] and for predictive maintenance [72].

### 4.3. Understanding the Latent Space of Representations

The latent space in which learnt representations reside can serve as feature spaces for downstream machine learning applications, including classifiers and other supervised predictors, for example, k nearest neighbours, softmax and fully convolutional layers. The analytics and inferences that can be made do not stop here, however, as there is a wealth of untapped potential that has yet to be exploited in many applications from domains such as geometry, information science and hybrid–human intelligence. This section will explore techniques that have been introduced in some of these domains.

#### 4.3.1. Latent Space Visualisation

The interpretation of latent space often requires subtle and implicit domain knowledge, for which human judgment is essential. However, dimensionality reduction techniques are often essential for visualising multi-dimensional latent spaces as humans have difficulty in reasoning about space beyond three dimensions. Common projection methods include t-distributed stochastic neighbour embedding (t-SNE) and principal component analysis (PCA). T-SNE is a non-linear technique that aims to match neighbours in the original space to those in the lower dimensional embedding. It is popular for exploring very high-dimensional data and with data with many embedding groups if the perplexity of the output projection is interpreted appropriately [73]. Uniform manifold approximation and projection (UMAP) is another non-linear technique that better preserves inter-cluster relationships. These non-linear algorithms highlight cluster structures but can obscure linear relationships among points. PCA is a linear transformation and so preserves linear relationships [74], which might be beneficial if further inferences can be drawn from the relative distances between embeddings.

**Figure 4:**
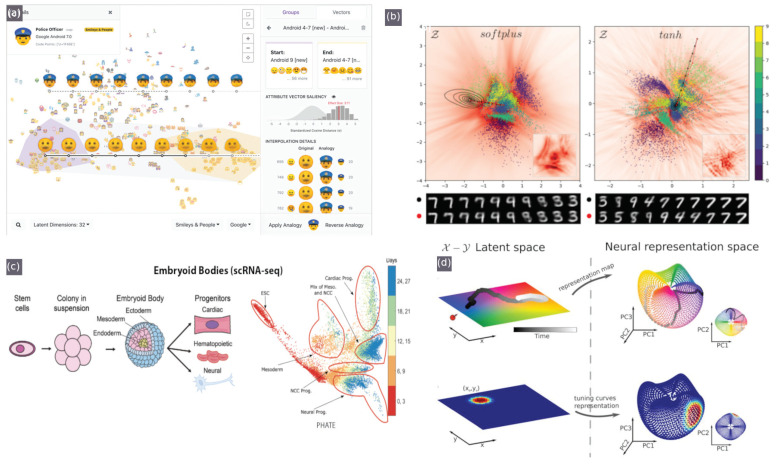
Latent space visualisation tools: (**a**) latent space cartography. Reproduced with permission [74]. (**b**) Generalised metric-inspired measures and measure-based transformations for generative models. Reproduced with permission [75]. (**c**) PHATE. Reproduced with permission [77]. (**d**) Manifold analysis for navigation tasks, where a navigating agent learns to predict the upcoming sensory observation, and the dynamical and geometrical properties are captured in a neural representation manifold. Reproduced with permission [76].

Recently, interactive tools for visualising latent space have been developed, initially focusing on a specific domain and a narrow set of tasks, and even more recently, such interactive elements have been compiled into integrated tools. Latent space cartography [74] seeks to guide users through a comprehensive workflow that supports tasks common to latent spaces across various input data types and RL algorithms. These tasks include changing the desired type and complexity of projection algorithms, querying, filtering and highlighting groups of embeddings and visualising the similarity of these groupings with attribute vector arithmetic [74] (shown in Figure 4a).

Transformed space, colourisation, textured plot overlays, contour maps (equidistant lines) and interpolation paths can help make sense of the measure and progression of change in relation to meaningful metrics [75] (as shown in Figure 4b) and can also be useful in navigation tasks [76] (as shown in Figure 4d).

#### 4.3.2. Multi-Task/Multi-Metric Correlation

Multi-task approaches jointly train a single network to perform multiple tasks, thereby sharing useful information among the tasks, which significantly improves their performances.

Even when tasks are assumed to be independent, similarities in the adjacent region/data surrounding objects/events can still induce knowledge sharing (inductive bias transfer) [78]. Even early statistical methods took advantage of this. Multivariate surveillance methods based on likelihood ratio tests in the presence of spatial correlations are effective in taking advantage of spatial correlations to provide faster and more accurate detection in bio and healthcare surveillance [78] and industrial process control [79]. Multi-task learning (MTL) makes use of a complementary loss function, i.e., the loss function sums the result of several sub-functions, each responsible for one or more tasks. The sheer existence of multiple tasks means that the loss function will not approach zero until all sub-functions are optimised, which causes the model to prefer the hypothesis that can solve all tasks simultaneously [80]. One challenge with this approach is weighting the sub-functions of the complementary loss function so that each sub-function’s contribution to the overall loss is balanced [81].

The sharing of information between tasks induced by MTL has led to it being used just to improve the performance of the primary task [82] even when just using auxiliary unsupervised tasks on unlabelled data [83]. Many multi-task metric learning (MTML) approaches learn a Mahalanobis distance parametrised by a positive semidefinite (PSD) matrix *A*, which facilitates the learning of a linear transformation *L* in feature space, since $A=L^{T}L$, to be applied so that all embeddings are mapped to one feature representation space that properly separates different categories for several tasks simultaneously. This methodology syncs well with the concept of sparse metric learning. If the principal components of input feature vectors $X_{i}$ are expected to be sparse, then applying the transformation vector, $\tilde{X_{i}}$, should ideally nullify columns containing noise to yield a feature vector with fewer dimensions and make learning less time-consuming and expensive [84]. This type of regularisation approach has also been shown to apply to generative/autoencoder networks [83] and GNNs [85]. The most appropriate architecture depends on the multi-task problem. For example, GNNs can encode the topological structure of multiple properties in a more natural way for applications such as molecular chemistry [85]. While generative models can generally capture more salient features within the data, the ground truth feature information is intractable at inference [83], whereas DML-based approaches can learn to encode FG information into certain metrics in feature space.

A common application on multi-task RL is detecting the subgroups that have similar characteristics in feature space. One method of doing this is to incorporate a clustering step (e.g., K-means) into the regression stage so that both grouping and sub-grouping tasks can be performed simultaneously [86]. A similar approach was taken in an FG building change detection application, where the authors of [87] adopt an encoder–decoder architecture and constrain the primary change detection task’s loss function with and an auxiliary semantic segmentation task to direct the model to better include building footprint detection error.

These techniques can also be adapted for FG analyses, as demonstrated by the authors of [88], who implement a regularised multi-task ordinal regression model with shared representation layers that encode task relatedness in such a way that allows regression of the progression of disease to be performed.

#### 4.3.3. Alternate Space Representation

Transformations that can be applied to the latent space that are key to mapping representations relative to auxiliary and expert-provided data are key to facilitating knowledge injection a posteriori to refine the metric space specifically to the observation query. These include geometry preservation techniques, such as using von Neumann divergence to measure the spread of certain metrics to produce non-isotropic overlays over latent space projections [89]. Such overlays have been well demonstrated by [75], as shown in Figure 4b and by [74].

Latent space can also be transformed into many alternate space representations beyond conventional Euclidean geometry, e.g., hyperbolic space with negative curvature, which can embed tree-like structures [90,91]. Such space representations can reveal data structure and patterns in a more intuitive form. For example, by fitting embeddings to a manifold [92], the local curvature at any point can be easily calculated and hence the divergence of embeddings with respect to each other can be known. In this way, manifold regularisation can take advantage of labelled and unlabelled information, which can be useful if there are missing data or in fine-grained tasks where sub-class details must be inferred. Many weakly/semi-supervised approaches are based on the manifold assumption, which means the sample points are concentrated upon a low-dimensional manifold instead of being filled in the whole feature space. Traditional low-dimensional manifolds such as IsoMAP, Laplacian eigenmaps, diffusion maps and local tangent space alignment (LTSA) approximate the geometric structure of data such that the local geometry is optimally preserved [93]. Manifold learning algorithms can be categorised as being a hybrid of metric learning, graph embedding and unsupervised learning. Each essentially takes points in metric space $p\in R^{r}$, and use a neighbourhood graph G and/or similarities between points in order to obtain an embedding in $R^{s}$, which can have reduced dimensionality because the inherent structure has been learned from the data unsupervised [94].

Traditional manifold learning algorithms assume that the embedded manifold is globally or locally isometric to Euclidean space. However, by breaking that assumption, some recent techniques have shown it to be advantageous to consider the curvature of the embedding manifold, i.e, to use geodesic distance rather than Euclidean distance. These techniques can achieve better stability and reduce the dimension of the general manifold [95]. The mathematics facilitated by manifold structures has been shown to better describe continuous change by excavating the curvature information of Riemannian sub-manifolds as well as distance metrics to uncover the intrinsic geometric structure of local patches in point clouds [95] and images [96]. The theory behind this is that Riemannian manifold $\left(M,\;g\right)$ is defined by a positive-definite inner product $g_{p}$ on the tangent space at each point $T_{p}M$, which enforces the manifold to be smooth. The inclusion of tensor Riemannian metrics in the loss function can therefore enforce several geometric relations among neighbourhoods of embeddings, e.g., regularisation of the angle at an intersection between any two points, optimisation of the surface integral or regularisation of the extrinsic and/or intrinsic curvature of the manifold itself. Riemannian metrics can be incorporated into a metric learning framework without requiring any modifications in the existing deep metric learning architecture by Riemannian optimisation (enforcing orthonormality constraints on parameter matrices as part of the loss function), as demonstrated by [96].

A number of techniques have been developed recently to derive structure from few representations. The authors of [97] have addressed the problem of defining distances between points on an unknown manifold while taking into account the intrinsic density following Fermat’s principle, also known as the principle of least time. Hessian regularised distance metric learning [98] is another example. The Hessian matrix or Hessian is a mathematical technique that can be used to describe the local curvature of a function of many variables in the form of a square matrix of second-order partial derivatives of a scalar-valued function, or scalar field. This can be advantageous in change regression applications where the Hessian can learn functions whose values vary linearly with respect to geodesic distance. The approach is especially useful in fine-grained change regression problems with few labelled pieces of information across the possible range of values, which can be expected due to the good extrapolating power, i.e., because the outputs of the functions contained in Hessian vary by linearity with the geodesic distance along the underlying manifold.

#### 4.3.4. Structured Representations

Structured data infer complex latent structure in data (it can be naturally clustered into sub-classes or organised based on class-specific properties) but often suffers from computational and capacity issues when dealing with large amounts of complex, high-dimensional data, e.g., sequences, trees, and graphs. RL generally focuses on the challenge of converting structured data to a vectorial representation in the first place, such that subsequent problems, e.g., similarity/distance estimation, become easy to solve [99]. However, some recent research has investigated engineering structured representations.

The manipulation of FG representations is a challenging problem, as FG details are difficult to capture. Most existing CD methods resort to discrete labels, which is generally only effective for expressing global changes and ignores the manipulation of fine details. One example solution to address this challenge in an FG expression manipulation application is to utilise structured latent codes and continuous expression labels [100].

Structure can also be derived from manifold representations. For instance, PHATE, a visualisation method that captures both local and global non-linear structure using an information-geometric distance between data points for predicting interactions between proteins and other biomolecules solely based on structure [77] (shown in Figure 4c). Information geometry preservation in metric learning has also been implemented similarly using Von Neuman entropy/divergence [89], and Infomax (an optimisation principle that maximises the average mutual information (MI) between different projections of data, where MI is the amount of information obtained about a random variable X by observing some other random variable Y) [101,102,103].

Another type of structured representation lies in a method we have discussed already, GDL. A series of translation-based methods have been proposed for knowledge graph embedding to project the nodes (also called entities) and the edges (also called relations) of the knowledge graph onto a continuous vector space [104]. The resulting graph embeddings are therefore structured and similar geometric transformations and Infomax principles can and have been applied to these embeddings to improve subgroup relatedness recognition [105]. Although the GNN-based model offers sparse representation learning capacity, it is limited by the specification of the graph structure design and it can be non-trivial to generalise it for latent space interpolation [106].

On that note, it is important to be aware that structured representations, whether based on handcrafted features or incorporated into deep networks, suffer from one drawback. They aggregate local information from the entirety of the input data, regardless of how relevant this information is to the recognition task. In practice, however, while certain regions contain semantic information that contribute to the target label, others naturally do not. Incorporating information from these uninformative regions, which can appear in many other categories, will typically yield fewer discriminative representations [107].

Another structured representation is evident in tensor representation learning. Tensors are generalisations of matrices to N-dimensional space. Aside from holding numeric data, like a vector does, tensors also include descriptions of the valid linear transformations between tensors, i.e., it is defined to change coordinates in a certain way under certain changes of variables and therefore isolates intrinsic geometric and physical properties from those that merely depend on coordinates. A multi-temporal hyperspectral remote sensing image change detection approach has been proposed by the authors of [97] to form a tensor-based information model of underlying features change, which optimises the organisation mode and maintains the integrity of constraints between different underlying features. The tensor model allows full use to be made of deep belief networks, support tensor machine and 3D-DWT wavelet texture extraction technology to improve the change detection accuracy [97].

## 5. Challenges, Comparisons, and Future Directions for Change Representation Techniques

The previous section details a number of techniques that have arisen from a diverse range of application domains to address challenges and leverage opportunities often specific to the traits of the data available/requirements of the application. In this section, we group some of these challenges under categories relating to requirements for adaptable real-time response, input data inconsistencies and model interpretability. Under each category, we discuss some recent approaches to these problems and offer some perspectives on trends in the uptake of some of these techniques towards addressing these problems.

### 5.1. Real-Time and Online Change Detection

Most CD applications require change detection to be performed in real-time, i.e., they require data to be processed sequentially and for change-points to be detected as soon as they occur or within a certain time window [78]. This can be considerably more challenging as retrospective offline techniques have the advantage of access to the data before and after the point to decide whether the data distribution has changed. This problem is known as quickest change detection (QCD) [16] and is common in applications such as manufacturing quality control and fall/incident detection in patient monitoring. Furthermore, these applications typically require the algorithms to be deployable on edge devices, which implies real-time processing with limited computation complexity. The more basic statistical methods excel in terms of computation time and hence are still relevant if the problem is not too complex, e.g., seasonal-trend decomposition and likelihood ratio statistics to detect the changes [78]. The segmentation approach used in graphical methods suffers here due to the high dimensionality of the output difference image/change map; although, real-time detection is possible if trained properly. Representation learning approaches can be quite favourable in comparison as good representations need to have low dimensionality by design and the greater recognition abilities of neural networks.

Another related field of research that deals with the challenge of applying deep learning to data on the fly is online learning, which requires new classes to be recognised at deployment. Continual learning or lifelong learning refers to the ability to continually learn over time by accommodating new knowledge while retaining previously learned experiences [46,108]. The catastrophic forgetting problem, mentioned in Section 4.2.1, is present, and with regards to FGCD, we identify the process of CD as being a key tool for continual learning in general. It has been demonstrated by [109] that detecting changes in dense RGB-D maps over the lifetime of a robot can aid in automatically learning segmentations of objects.

### 5.2. Change Detection on Heterogeneous Data

There are many challenges associated with heterogeneous data sources, i.e., the input data for each of the tasks might contain missing values, the scale and resolution of the values is not consistent across tasks and the data contain non-IID instances.

A methodology that may be applied to non-visual data/a hybrid of visual and non-visual data is to first convert the non-visual data so that it can be viewed as an image (e.g., activity data from wearable sensors can be visualised in the form of a density map that uses different colours to show varying levels of activity [110,111]) and then proceed with image-based techniques. However, the way that the data are encoded into image form can influence the results as most convolution-based networks are not permutation invariant.

Another technique that is useful for continuous variables is kernelisation, which is a technique for replacing input with a kernel, a function that is symmetric and positive definite. By virtue of positive-definiteness, the kernel function allows us to transform our input to a domain where we can solve problems more-efficiently and then use tricks discovered in that domain in the original domain. A classic example of this is in use in support vector machines for non-linear regression. Furthermore, kernelisation can allow us to represent the desired output on ordinal, interval or ratio scales, which may be more useful in some applications. A number of papers have proposed techniques for performing regression with DML using kernelisation [84,112,113].

Sparse compositional metric learning was proposed by [114]. It learns local Mahalanobis metrics for multi-task/multi-class data on sparse combinations of rank-one basis metrics. Sparse metric learning pursues dimension reduction and sparse representations during the learning process using mixed-norm regularisation, which results in much faster and efficient distance calculation [115]. This concept also allows learning on sparse and unbalanced data. Much of this type of research took place before the advent of deep learning, and therefore, there is an opportunity for these techniques to be applied to deep networks.

### 5.3. Interpreting Change from Representations

Explainable artificial intelligence (XAI) refers to AI that produces details or reasons to make its functioning clear or easy to understand. These principles can be applied to the interpretation of latent spaces in RL to assist the evaluation of models, help explain model performance, and more generally aid understanding of what exactly a model has “learned” [74].

For example, some papers use discriminative clustering in latent spaces to decide whether different classes form distinct clusters; however, if we want to explore the latent space further to understand the underlying structures in the data, we need visualisation tools [74]. From these analyses, one may discover useful metrics that may be exploited, e.g., clusters in the latent space may be found to reflect that distance between the same words from embeddings trained on different corpora signifies a change in word meaning in certain contexts [116].

#### 5.3.1. Trialling Different Visualisations

A key decision to be made when interpreting latent space, or indeed during any data analysis, is whether the identified features represent true features of the underlying space rather than artefacts of sampling. A common example of misreading projections of latent space is with t-SNE, where conclusions are drawn without trialling different parameters of the projection algorithm such as the perplexity that needs to be tuned in proportion to approximately the number of close neighbours each point has in order to balance attention between local and global aspects of the data.

Persistent homology (PH) is a method for automating this type of procedure by computing the topological features of a space at different spatial resolutions. [117]. Topology provides a set of natural tools that, amongst other things, allows the intrinsic shape of the data to be detected using a provided distance. As well as being integral to geometric deep learning, the field of research known as topological data analysis (TDA) has gained popularity in recent years using these tools to quantify shape and structure in data to answer questions from the data’s domain [118].

While homology measures the structure of a single, stagnant space, persistent homology watches how this structure changes as the space changes. Each data point is plotted on a persistence diagram as a pair of numbers (*a*,*b*) corresponding to its birth diameter and death diameter (i.e., the test instances at which a feature was first seen and last seen). More persistent features appear far away from the diagonal on a persistence diagram, are detected over a range of spatial scales and are deemed less likely to be due to noise or a particular choice of parameters. Persistent homology is just one form of topological signature that can show a great deal of information about a set of data points such as clustering without expert-chosen connectivity parameters and loops and voids that are otherwise invisible [118]. PH has been used for the detection of changes in land cover [119], structural changes in time-varying graphs [120] and brain morphometry [121].

#### 5.3.2. Explainable Change Detection

Once a change is detected and determined significant, additional analyses are required to explain the reason change that occurred. This problem is formally known as change analysis (CA), a method of examination beyond CD to explain the nature of discrepancy [122]. This field of research has explored methods for detecting and explaining change in time series data [123], remote sensing data [124] and diagnosis prediction. CA methods can be classified as being parametric or non-parametric. The former is where a parametric functional form is explicitly assumed to model the distribution.

CA falls in the category of unsupervised learning. Most existing FGCD methods spend efforts on mining global and/or regional discriminative information from training data themselves. For example, state-of-the-art methods learn to identify discriminative parts from images of FG categories through the use of methods for interpreting the layers of convolutional neural networks, e.g., Grad-CAM (gradient-weighted class activation mapping) [125] and LIME (local interpretable model-agnostic explanations) [126]. However, the power of these methods is limited when only few training samples are available for each category. To break this limit, possible solutions include identifying auxiliary data that are more useful for change detection specific to each class and also better at leveraging these auxiliary data [127]. Recently, there has been some interesting progress in applying Grad-CAM techniques to metric-learnt representations by [128], who generate point-to-point activation intensity maps between query and retrieve images to show the relative contribution of the different regions to the overall similarity. Not only can this technique produce better activation maps, but they are also instance-specific, which we believe is ground-breaking for FG analyses.

The incorporation of causal reasoning into ML research has also been gaining popularity in recent years. Traditionally, focusing on probabilities and correlation, ML and statistics generally avoid reasoning about cause and effect. However, this teaching has been criticised as being detrimental to the potential understanding, which can be gained from techniques such as counterfactual explanations, a specific class of explanation that provides a link between what could have happened had input to a model been changed in a particular way [129]. Causal representation learning is a by-product of this research activity, and its applications have reached explainable CD [130,131].

#### 5.3.3. Theoretically Grounded Change Detection

Theoretical research interests related to modelling complex systems require, not only for system dynamics to be captured and detected by a model but also for these changes to fit with what we currently understand about the system, e.g., to comply with the equations we have derived. Incorporating domain knowledge can be hugely advantageous as the theoretical model provides guidance with which an effective model is supposed to follow; it helps an optimised solution to be more stable and avoid over-fitting, it allows training with less data, it would be more robust to unseen data, and thus it is easier to be extended to applications with changing distributions [132]. However, this type of approach is only applicable to problems that have been studied extensively, as explaining the origin of change in terms of individual variables is generally a tough task unless the variables are independent.

Applications where theoretically grounded CD has been implemented include climate change [133] and dynamic systems [11]. These works implement techniques related to knowledge injection discussed in Section 5.3.4. Generally, they use an architecture based on graph networks to incorporate prior knowledge given as a form of partial differential equations (PDEs) over time and space. These PDEs can comprise very sophisticated mathematics, e.g., Lagrangian [134] and Hamiltonian mechanics [135].

#### 5.3.4. Latent Space Alignment

Latent space visualisations can seem arbitrary and not very meaningful when the dimensions of projections of the latent space are not aligned/scaled to important metrics specific to the application.

The performance of the RL crucially determines the type and performance of the algorithm for delineating the separation between feature sets to a manageable number of dimensions. However, techniques such as sparse metric learning can also be applied to further reduce the dimensionality of the embedding representation. Methods for sparse metric learning include mixed-norm regularisation across various learning settings to whittle down latent dimensions that do not consistently contribute to producing distinguishable representations [115] and sparse compositional metric learning, which learns local Mahalanobis metrics on sparse combinations of rank-one basis metrics [114].

Expressing representations in relation to familiar metrics can be useful in the visual evaluation of model performance by highlighting cases where there was an underlying pattern not explained by the primary tasks (e.g., scene change detection) of an RL approach but due to some other ancillary variables (e.g., weather). This may be applied to RL to reveal the interactions of background/ancillary variables by these variables to the axes of latent space/manifold visualisations, i.e., it may be useful to be able to tell why an object was classified to belong to a particular sub-class through observation of where that object lies on a space projection. We propose that by using interactive latent space cartography, which allows custom axes and colours according to selectable variables of interest, such relationships may become easily revealed. Moreover, it will help make the resulting visualisation of the embedding space more meaningful for the application. Such a visualisation of the feature space that takes into account known priors (e.g., weather conditions) has been shown to be useful in further refining the predictions at runtime [127].

If such auxiliary variables are known before inference, it may also be useful to narrow down the CD results to instances that are more likely in light of this new knowledge. This is known as knowledge injection and has been implemented in different ways depending on the type of RL. Auxiliary knowledge can be encoded as sparse input to metric learning techniques, as rules for more accurate relation extraction in generative approaches [63], or to predict missing links in knowledge graphs [136,137]. Alternatively, a clustering algorithm, e.g., k-means clustering, could be formulated taking as input the salient background variables and outputting a function that maps the latent space to valid classifications, thus maximising the inter-class variance in FG applications.

## 6. Overview

CD, the problem of identifying changes in data, constitutes an extensive body of research as many applications are requiring efficient, effective algorithms for reliably detecting variation. There are many families of CD algorithms that are suitable for different applications. These include approaches that quantify change statistically, graphically, and algorithmically and each offer their advantages in the face of the challenges of FGCD reviewed in this article.

This article has focused on RL solutions, which are a family of methodologies that exploit the effectiveness of DL in learning representations from little data. In general, representations occupy a unified feature space to connect heterogeneous objects, thereby achieving fusion and calculation between different types of information. The feature space can be transformed, projected and visualised and several novel techniques have been proposed in recent years, which have benefits to FGCD problems.

Many of these techniques, which we have surveyed in this article, can be incorporated in an additive fashion, i.e., a representation learner can learn transformations to feature space with multiple functionalities, including regularisation for sparse metric learning and or multi-task learning, kernelisation for ordinal regression, geometry preservation for maintaining intrinsic structure and information-theoretic feature selection and projection for aligning to known prior understandings. These endeavours aim to provide deeper, more interpretable FG predictive capabilities so that important change-points can be detected more reliably in applications such as patient monitoring and environmental monitoring. We believe that this is an important pursuit as producing outputs from artificial intelligence that we can trust involves revealing the reasoning behind predictions in terms of metrics/relations we understand. There is a lot of activity towards producing representations that incorporate relational information and more work to do in learning representations that can communicate the most pertinent FG information for any given query to be useful and trustworthy in practical change detection applications.

A gap in the research that we have touched on in this article is how the mapping element of RL may be exploited in situations where the salient features in arbitrary CD problems vary dependent on the intrinsic structure of data and auxiliary background variables. If the distribution of representations per any given variable may be projected and also observed with the use of interactive visualisation tools, then the influence of each variable on the CD task may be better understood. This review has observed some developments in this direction, although most still use unsupervised clustering techniques and deep learning, multi-task and fine-grained recognition concepts can be further exploited in this field. For example, there is an opportunity for the sampling strategy of a few shot learning methods to better exploit change metrics, and for prior information on salient background, variables may be exploited at the inference stage of the RL approach, possibly by taking advantage of human intuition with hybrid human–machine intelligence.

Another opportunity is for the latent space/manifold of RL to be used as a means of calibrating deep learning models. Since many manifold learning methodologies use the smoothness of a latent manifold as a means of regularising model response in a few-shot learning context, the adjustment of points on the manifold may theoretically be used to correct performance at deployment, i.e., if the model is observed to deviate from ground truth, the point of deviation may be adjusted, and the effect of the adjustment be propagated around the surrounding neighbourhood on the latent manifold in order to regularise the model. The challenges still present lie in determining what weight to give adjustments and over how wide an area of the manifold the adjustments should propagate, taking into account unreliability introduced by human interference with the model. Some knowledge from other domains such as calculus of variation and information geometry, which have already been integrated into some of RL techniques in this review, may be useful in achieving this goal.

## Abbreviations

The following abbreviations are used in this manuscript:

ADAS	Advanced Driver Assistance System
CA	Change Analysis
CD	Change Detection
CNN	Convolutional Neural Network
CR	Change Representation
CUSUM	Cumulative Sum
DL	Deep Learning
DML	Deep Metric Learning
DNN	Deep Neural Network
DWT	Discrete Wavelet Transform
FG	Fine-Grained
GAN	Generative Adversarial Networks
GDL	Geometric Deep Learning
GNN	Graph Neural Network
IID	Independent and Identically Distributed
QCD	Quickest Change Detection
t-SNE	t-Distributed Stochastic Neighbour Embedding
LTSA	Local Tangent Space Alignment
MAML	Memory Augmented Meta Learning
MTL	Multi-Task Learning
MTML	Multi-Task Metric Learning
PCA	Principal Component Analysis
PELT	Pruned Exact Linear Time
PH	Persistent Homology
PSD	Positive Semi Definite
RL	Representation Learning
SPC	Statistical Process Control
TDA	Topological Data Analysis
UMAP	Uniform Manifold Approximation and Projection
VAE	Variational AutoEncoder
XAI	eXplainable Artificial Intelligence
